# Organ‐specific low‐dose assessment in pediatric radiotherapy using nanodot OSL and NTCP modeling: Application to medulloblastoma

**DOI:** 10.1002/acm2.70444

**Published:** 2026-01-02

**Authors:** Meriem Tantaoui, Mustapha Krim, Fatimzahra Chehab, El Mehdi Essaidi, Mohammed Reda Mesradi, Abdelkrim Kartouni, Souha Sahraoui

**Affiliations:** ^1^ Subatomic Research and Applications Team, Laboratory of the Physics of Condensed Matter (LPMC‐ERSA), Faculty of Sciences Ben M'Sick Hassan II University Casablanca Morocco; ^2^ Laboratory of Sciences and Health Technologies High Institute of Health Sciences (ISSS) Hassan I University Settat Morocco; ^3^ Mohamed VI Center for the Treatment of Cancers of the IBN ROCHD University Hospital Center, Faculty of Medicine and Pharmacy Hassan II University Casablanca Morocco

**Keywords:** 3D‐CRT, anthropomorphic phantom, low doses, medulloblastoma, NTCP, OSL dosimetry, pediatric radiotherapy, risk modeling, VMAT

## Abstract

**Background:**

In pediatric radiotherapy, controlling low‐dose exposure is a major challenge, particularly in the treatment of medulloblastoma, where the long life expectancy of young patients makes optimization of radioprotection crucial.

**Purpose:**

This study provides a comprehensive and quantitative assessment of low‐dose exposure to normal tissues across the entire radiotherapy workflow, with a particular emphasis on the contribution of imaging procedures (planning computed tomography (CT) and repeated cone beam computed tomography (CBCT) acquisitions) in addition to therapeutic irradiation. The cumulative impact of these steps is evaluated together with the associated risks using Normal Tissue Complication Probability (NTCP) modeling.

**Methods:**

A pediatric anthropomorphic phantom was equipped with optically stimulated luminescence (OSL) dosimeters to measure doses delivered to normal organs during the planning CT, 15 CBCT scans for positioning, and simulated treatment plans with different radiotherapy techniques. NTCPs were calculated for critical organs based on the dosimetric data from imaging and treatment planning.

**Results:**

Each imaging acquisition delivered between 3 and 7 mGy per organ, with the cumulative dose from CT and CBCT remaining consistently below 0.5 Gy, representing less than 1% of the prescribed therapeutic dose. Although small compared to therapeutic irradiation, this contribution remains biologically relevant when considering cumulative low‐dose exposure in pediatric patients. Dosimetric comparison of treatment techniques showed that volumetric modulated arc therapy (VMAT) significantly reduced both the mean organ dose and the NTCP compared to three‐dimensional conformal radiation therapy (3D‐CRT): heart dose decreased from 16 Gy (NTCP 30%) to 6.6 Gy (NTCP 0.4%), and thyroid dose from 27.1 Gy (NTCP 12%) to 8.7 Gy (NTCP < 1%). Some organs, such as the brain and chiasma, remained highly exposed, reflecting the dosimetric constraints of comprehensive tumor coverage.

**Conclusion:**

By systematically quantifying and integrating imaging doses with therapeutic irradiation, this study underscores the often underestimated role of imaging in cumulative exposure during pediatric craniospinal irradiation. The results provide a solid foundation for tailoring radioprotection strategies and emphasize the need to reconsider planning and follow‐up protocols to deliver safer and more targeted treatments for children.

## 1 INTRODUCTION

Radiotherapy is an essential modality in the treatment of pediatric medulloblastoma, a common malignant pediatric tumor with cranial‐spinal involvement. Its effectiveness relies on the precise delivery of a therapeutic dose to the target volume while minimizing irradiation of surrounding normal tissue. However, young patients have increased sensitivity to radiation due to their rapid cell growth and prolonged life expectancy, which puts certain organs at increased risk of late side effects, including secondary cancers and functional complications ^1^, ^2^, ^3^, ^4^, ^5^, ^6^.

Exposure of normal tissue is not limited to the radiotherapy treatment phase. It results from the accumulation of doses from different stages of the treatment pathway, including: Dosimetric CT (Computed Tomography), used for treatment planning; CBCT (Cone Beam Computed Tomography) imaging for patient repositioning; and therapeutic irradiation itself, depending on the treatment modalities chosen: Intensity‐Modulated Radiation Therapy (IMRT), Volumetric‐Modulated Arc Therapy (VMAT) and, Three‐Dimensional Conformal Radiation Therapy (3D‐CRT), and so on.^7^. Each step contributes to the cumulative exposure of normal tissues, making accurate quantification of the absorbed dose essential to understand its impact better and optimize clinical practices ^8^, ^9^, ^10^.

In this context, optically stimulated luminescence (OSL) dosimeters provide a reliable and accurate method for measuring the absorbed dose in situ within a pediatric anthropomorphic phantom, thereby simulating real‐life irradiation conditions.^11^, ^12^, ^13^ In addition, the estimation of risks associated with this exposure can be carried out via dose–response models to quantify the potential for the development of secondary cancers or other radiation‐induced complications. Furthermore, modeling tools such as the Normal Tissue Complication Probability (NTCP) and the Equivalent Uniform Dose (EUD) provide valuable frameworks to assess the probability of radiation‐induced toxicities and to integrate heterogeneous dose distributions into clinically meaningful metrics. The EUD can be calculated according to Niemierko's formalism:

(1) $$EUD={\left(\sum v_{i}D_{i}^{a}\right)}^{\frac{1}{2}}\;$$

where $D_{i}$ is the dose delivered to the subvolume v and a is a tissue‐specific parameter. Based on the EUD, the NTCP can be estimated using the sigmoidal dose–response relationship:

(2) $$NTCP=\frac{1}{1+{\left(\frac{D_{50}}{EUD}\right)}^{4\gamma_{50}}}$$

where $D_{50}$ is the tolerance dose for a 50% complication probability and γ_50_ ​describes the slope of the dose–response curve. These concepts offer deeper insight into the balance between tumor control and normal tissue sparing, and their inclusion enhances the relevance of risk estimation in pediatric radiotherapy.^4^, ^5^, ^14^.

The objective of this work is to quantify the exposure of normal tissues throughout the patient pathway in radiotherapy for pediatric medulloblastoma, using a pediatric anthropomorphic phantom, allowing a realistic simulation of clinical conditions. OSL dosimeters were used to measure the absorbed dose in various critical organs at each stage of treatment. Risk modeling was applied to assess the potential impact of measured exposures on the occurrence of late side effects, using different 3D‐CRT radiotherapy techniques in two different phantom positions, as well as VMAT. This study aims at the quantitative evaluation of the dose due to irradiation of normal tissues in pediatric radiotherapy and to propose the radiotherapy pathway that most limits exposure outside the treatment field, thus reinforcing the principles of radioprotection adapted to children.

## 2 MATERIALS AND METHODS

The dosimetric assessment was performed by replicating the typical pathway of a pediatric patient receiving radiotherapy for medulloblastoma. Three key steps were considered to quantify normal organ exposure:

1. Dosimetric CT: Acquisition of computed tomographic images for treatment planning.
2. Positioning and CBCT imaging: Verification of the patient's position before each treatment session.
3. Therapeutic irradiation: Planning and administration of the prescribed dose were performed according to three planes: two 3D‐CRT planes performed in two different positions: the first plane using the 3D‐CRT technique but in the prone position (3D‐CRT‐PP), the second plane performed in a supine position (3D‐CRT‐SP), and a third plane using the VMAT technique in the supine position. All dosimetric measurements were performed on a pediatric anthropomorphic phantom with immobilization using a headrest system and a custom vacuum mold. The phantom is equipped with optically stimulated luminescence (OSL) dosimeters, allowing precise quantification of the doses absorbed by normal organs during the imaging stages, focused on in the planning CT and during the CBCT. The dose to normal organs during treatment was estimated using the treatment planning system (TPS), as such evaluations are estimated during the treatment planning phase.

### 2.1 Pediatric phantom

**FIGURE 1:**
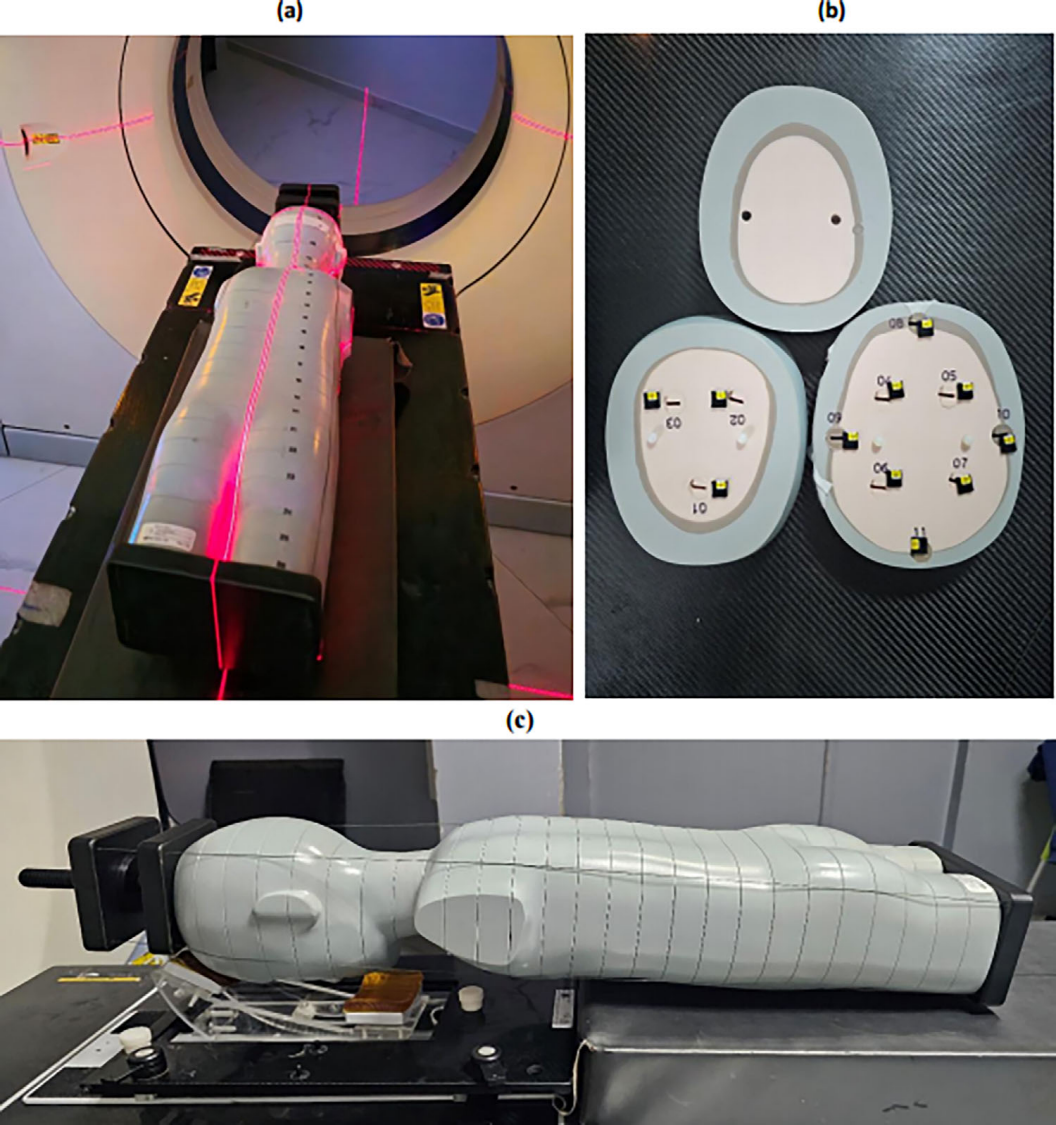
Images of the phantom (a) in CT planning (b) Sections of the cervical part of the phantom with OSL (c) positioning of the phantom with the means of restraint dedicated to repositioning during radiotherapy treatment planning.

In this study, we used an anthropomorphic phantom called Laurent that provides a realistic anatomical representation of a 5‐year‐old children's body child's body for the accurate assessment of absorbed radiation dose during medical examinations. The phantom is usually made from materials equivalent to human organs in terms of composition and density, including internal organs such as the heart, lungs, liver, kidneys, etc. This 5‐year‐old child phantom (Figure 1a–c) is the ATOM 705‐D model, CIRS (Computerized Imaging Reference Systems, Norkfolk, Great Britain), measuring 110 cm, 19 kg in weight, and 14 × 17 cm in thoracic dimensions. The anthropomorphic phantom, specifically designed with dedicated slots for the insertion of nanodot dosimeters, is cut into 26 slices (sections). The sections of the phantom allow accurate placement of 86 nanoDots within the phantom (Figure 1b), ensuring precise assessment of internal doses. The phantom was positioned according to standard clinical practices on the dosimetric scanner table and the linear accelerator treatment table, replicating real‐life treatment conditions throughout the pediatric patient pathway treated for medulloblastoma.^11^, ^15^, ^16^ Quality controls for reader accuracy and variability are described below.

### 2.2 Optically Stimulated Luminescence Dosimeter (OSLD) and reading system

Absorbed dose measurement was performed by the nanoDot OSLD and the *InLight MicroStar OSL reader* (Landauer, Inc., Glenwood, IL). The dosimeter contains a 5.0 mm diameter aluminum oxide disc with carbon with a density of 1.03 g/cm^3^. The exposure of these optically stimulated luminescence dosimeters (OSLD) was digitized by inserting them into the *MicroStar reader*. The reading was performed using *MicroStar* 5.2.0.0 software. The unit used according to the international system is mGy. The sensitivity of this system is 0.7 mGy. In the first reading step, bleaching of the OSLD was performed with the pocket annealer. In this work, a verification test of this step was performed for each nanoDot of the 119 OSLs used for dose estimation for the 3 steps of the pathway, and others for calibration to ensure the absence of radiation dose from a previous study.^17^, ^18^, ^19^, ^20^, ^21^, ^22^

### 2.3 OSL dosimetry and calibration protocols in CT and CBCT

The measurements were performed with nanoDot OSLD (Landauer), containing a carbon‐doped alumina disc. The MicroStar reader allowed accurate reading of the signal after irradiation. A rigorous calibration protocol, specific to each modality (CT, CBCT, RT), was applied: For imaging, the calibration used a reference ionization chamber, with dose/count conversion corrected by beam quality, linearity, angle, and fading factors. The calibration followed the AAPM TG‐191 protocol ^19^.

### 2.4 Measurement uncertainty

The total uncertainty of the absorbed dose was estimated by integrating all sources of error related to calibration and reading, using uncertainty propagation methods more robust than simple quadratic addition. This approach provides a realistic and reliable estimate of the standard deviation associated with each dosimetric measurement.^19^, ^23^

### 2.5 Planning parameters

The acquisition of a dosimetric CT and CBCT was carried out respecting the restraint conditions used in radiotherapy in order to ensure optimal reproducibility of the positioning.^24^, ^25^, ^26^ The orientation of the patient in imaging, whether prone or supine, only marginally affects the absorbed dose since these imaging modalities rely on an isotropic rotation around the body. Furthermore, as part of the treatment planning of medulloblastoma, a whole‐body dosimetric CT is systematically carried out in order to guarantee exhaustive anatomical delineation and to optimize the accuracy of the dose calculation.

#### 2.5.1 At the level of planning, CT scan

The entire ATOM phantom was scanned using a GE LightSpeed Pro 16 planning CT scan and the standard pediatric protocol for children mentioned in the literature, using the restraints used in medulloblastoma radiotherapy.^25^, ^26^ The acquisition parameters are listed in Table 1. The phantom was centered using lasers by placing the CT beads at head level, as in a protocol intended for medulloblastoma planning. It was scanned from the skull (Slice 1) to the last cut of the phantom (Slice 26). The CT images were reconstructed with a thickness of 1.25 mm.

**TABLE 1:** Scan parameters of the full‐body planning CT scan protocol used in this study.

Type of acquisition	Number of images	Thickness (mm)	Scan Field of View (SFOV)	kV	mA	Time of acquisition	CTDIvol (mGy)	Dose Length Product (DLP) (mGy*cm)
Helical Full 0.6 s	738	1.25 mm	Large	120	90	16.36	5.3	352.6

#### 2.5.2 At the level of CBCT

The accelerator imaging system, using the onboard imaging system of a Versa HD linear accelerator (Elekta), was used to scan the Laurent phantom (Figure 1a) at the cervical and thoracic regions (considering the two iso‐treatments used in the medulloblastoma planes) in full fan mode with x‐ray source parameters with a peak voltage value of 120 kVp, a tube current of 80 mA, and an exposure time of 25 ms. The beam collimation was 20.6 cm, producing an axial dimension of a reconstructed image of 16 cm. Other CBCT parameters included a spot size of approximately 0.4 to 0.8 mm, a source‐image distance of 100 cm, a total number of projections of approximately 650–700, a field of view of 25 × 25 cm^2^, and bow‐tie filtration.

#### 2.5.3 At the treatment level

**FIGURE 2:**
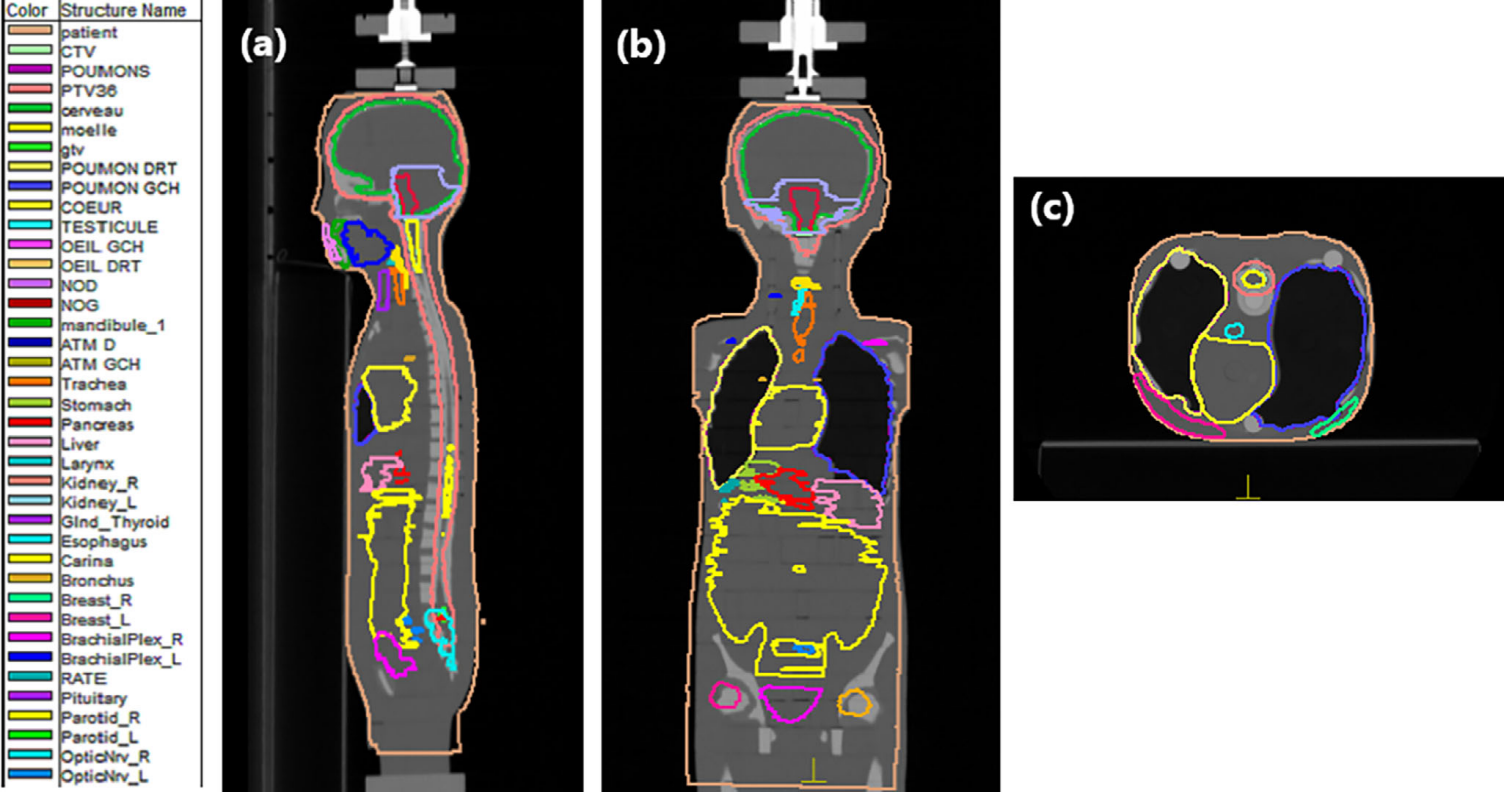
DICOM images of the Laurent Phantom (a) Frontal section, (b) Sagittal section, (c) Transverse sections with delineation of volumes.

DICOM images of the ATOM phantom (CIRS, Norfolk, VA, USA) were acquired using a GE LightSpeed Pro 16 computed tomography (CT) scanner. Two positioning options were available: supine and prone, as shown in Figure 2. These images were then exported to the Monaco v5.00 Treatment Planning System (TPS) for delineation of anatomical structures and optimization of treatment plans.

Target volume delineation was performed according to contouring guidelines for pediatric cancers^27^. The cranial PTV was defined by adding a 4 mm margin around the cranial CTV, while the spinal PTV was delineated with an equivalent 4 mm margin around the spinal CTV. A PTV boost was also defined, corresponding to a 4 mm extension around the CTV boost, the latter representing the entire posterior fossa without the brainstem.

All organs at risk (OAR) were identified and segmented, comprising a total of 20 structures distributed at the cranial and spinal levels, to optimize treatment planning and limit their radiation exposure.

#### 2.5.4 Planning methodology and irradiation techniques

Treatment planning was performed using two dose calculation algorithms: Collapsed Cone Convolution (CCC) for three‐dimensional conformal radiotherapy (3D‐CRT) and Monte Carlo for volumetric arc therapy (VMAT).

Three treatment plans were established, based on the phantom position and the irradiation technique: 3D‐CRT‐PP, 3D‐CRT‐SP, and VMAT‐SP.^7^, ^27^ The prescribed dose was 36 Gy in 20 fractions for the cranial and spinal PTV, while the boost PTV received an additional dose of 18 Gy in 10 fractions. These treatment plans were designed to irradiate the target volumes while minimizing the dose to normal organs during radiotherapy, to mitigate radiation‐induced complications, and preserve the children's future quality of life.

#### 2.5.5 Parameters and optimization of beams in 3D‐CRT

The 3D‐CRT‐PP plan was designed using two lateral fields at the cervical level, with the collimator angulation adjusted to achieve a homogeneous junction with the posterior field (gantry = 0°) covering the thoracic, abdominal, and pelvic regions. The treatment isocenter was placed at the thoracolumbar junction. 6 and 18 MV beams were used to ensure homogeneous dose distribution throughout the tissue depth.

The 3D‐CRT‐SP plan follows a similar approach, but with a modification to the posterior field, where a single posterior beam is used to irradiate the spinal PTV, with the gantry angle set at 180°. For both 3D‐CRT plans, the tumor boost was planned using two lateral fields of 6 MV energy. Several optimization strategies were implemented to improve tumor coverage and minimize OAR exposure, beam energy mixing (6 MV/18 MV) to optimize dose distribution, the “field‐in‐field” technique to homogenize the dose within the cranial and spinal PTV, and then ballistics (beam geometry, beam trajectory) optimization to minimize the dose in out‐of‐field areas.

#### 2.5.6 Optimization and ballistics in VMAT

For the VMAT‐SP plan was performed with 6 MV photons, an optimized irradiation protocol was implemented with a three‐arc fractionation: a full arc (360°) for cranial irradiation receiving 36 Gy, two 60° arcs covering the posterior region of the phantom, targeting the spinal portion of the PTV receiving 36 Gy, and a posterior half‐arc dedicated to boosting the PTV irradiation. All treatment plans were optimized to ensure homogeneous tumor coverage, with a strict constraint ensuring that at least 95% of the prescribed dose covered the entire PTV. At the same time, OARs were spared as much as possible, respecting clinically tolerable dose limits.^6^, ^28^, ^29^

### 2.6 Modeling the risks associated with irradiation of normal tissues

**FIGURE 3:**
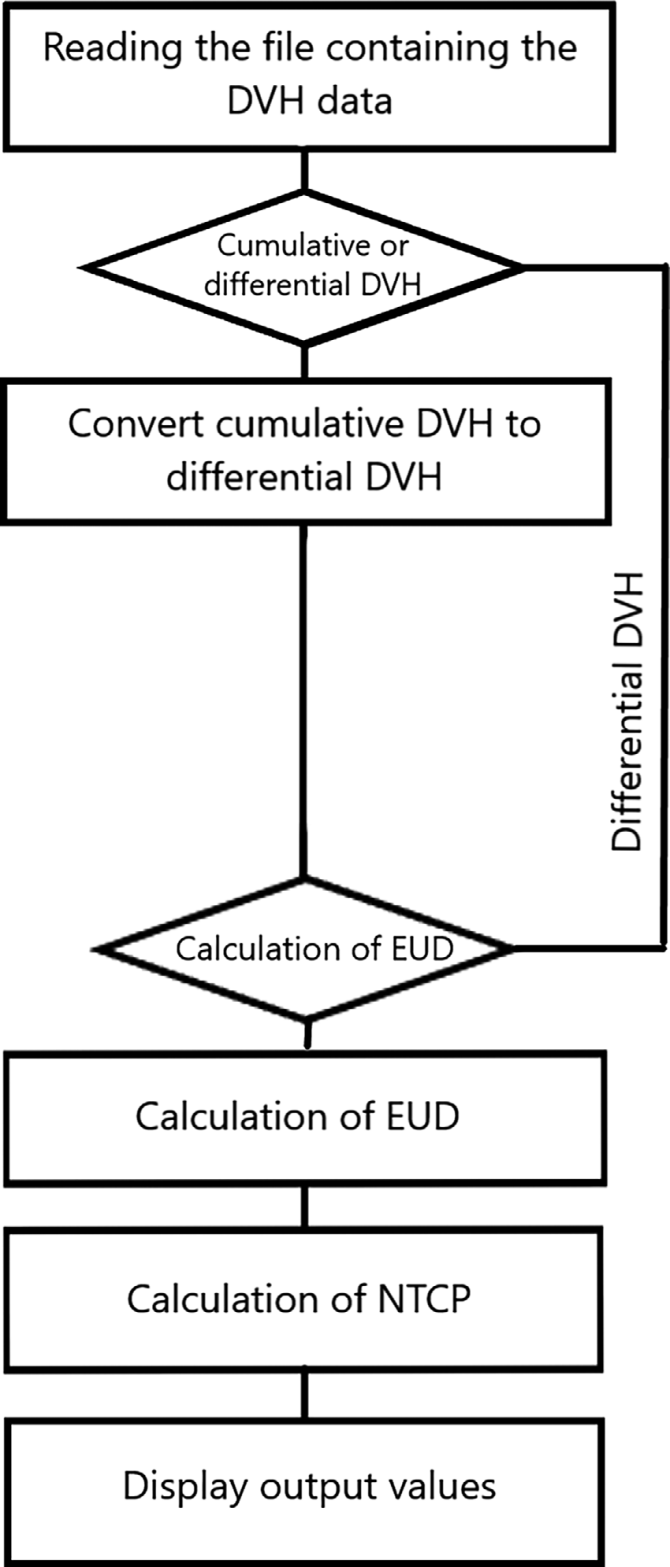
Python process diagram for calculating EUD and NTCP values.

The risk assessment of late complications induced by irradiation was performed by dose‐response models recognized in the literature. The approach used is based on tissue complication probability modeling (NTCP) equations^30^, ^31^, ^32^, ^33^, ^34^implemented using Python scripts. The NTCP calculation was performed using dose‐volume histograms (DVH) extracted from the treatment plans, allowing a detailed assessment of dose distribution in organs at risk (OARs). DVH extraction and analysis were automated using scientific libraries such as NumPy, SciPy, and Pandas, ensuring rigorous quantification of tissue response based on partially or fully irradiated volumes. These tools made it possible to implement probabilistic late toxicity models and assess dose effects on normal organs. The algorithmic implementation in Python ensured a quantitative and reproducible approach, reinforcing the reliability of the analyses. To better illustrate the methodology used, a Python process diagram detailing the steps involved in calculating Equivalent Uniform Dose (EUD) and NTCP values has been included in Figure 3. This representation visualizes the computational logic and ensures methodological transparency in the assessment of late side effects.

These results provide an essential basis for optimizing treatment protocols and reducing risks associated with pediatric irradiation by integrating advanced tools for predictive assessment of late side effects.

## 3 RESULTS

### 3.1 Low‐dose estimates in CT planning and CBCT

#### 3.1.1 At the level of CBCT Calibration factor at planning CT scan

**FIGURE 4:**
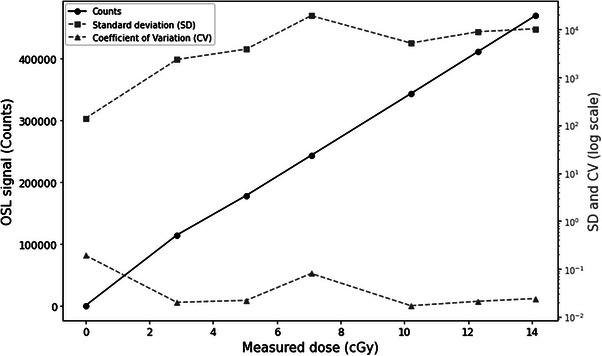
Calibration curve of OSLs irradiated with x‐ray gamma rays at the planning CT scan for different dose rates.

The data obtained from the counts and the absorbed doses show a direct, although not perfectly linear, relationship between the absorbed dose and the measured counts, illustrated in Figure 4. Counts increase with increasing dose, illustrating a sensitive response of OSLs to varied dose levels. For example, at a dose of 2868 cGy, the counts were 114 861 742, with a standard deviation of 2 351 959 and a coefficient of variation (CV) of 0.020, indicating high measurement accuracy at this dose level. In contrast, at a dose of 7077 cGy, the counts were 243 459 547 with a standard deviation of 19 356 615 and a CV of 0.080, showing higher relative variability of the measurements. The average calibration factor, calculated from dose/count ratios, is 2.937 × 10^−^⁵ cGy/count. This calibration factor makes it possible to convert the counts measured by the OSL into dose by taking into consideration the correction factors, as illustrated in the calibration section of the OSLs, facilitating the estimation of radiation dose during this planning CT scan procedure.

#### 3.1.2 Calibration factor at CBCT

**FIGURE 5:**
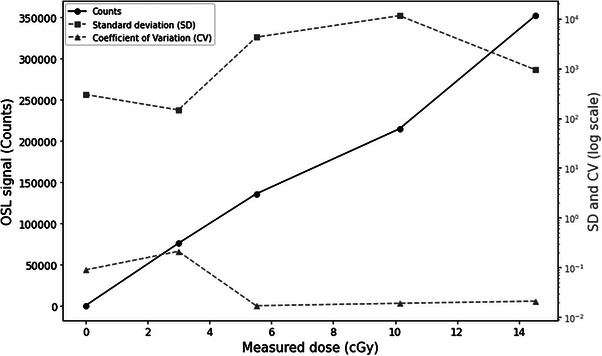
OSL calibration curve for CBCT dose measurements.

The calibration of optically stimulated dosimeters (OSLs) allowed the determination of calibration factors for doses between 0 and 14 cGy, as illustrated in Figure 5. The results show a calibration factor of 4.77 × 10^5^ cGy/count, which means that each count corresponds to a dose of 4.77 × 10^5^ cGy. In addition, the inverse calibration factor, representing the number of counts per unit dose, is 20957.66 counts/cGy. These factors are essential to ensure accurate conversion of measured counts to administered dose, thus enabling precise and reliable dosimetry.

#### 3.1.3 Evaluation of doses to organs

Dosimetric measurements performed using OSL dosimeters on the pediatric anthropomorphic phantom allowed the assessment of absorbed doses by different organs during the planning CT scan and a single CBCT acquisition. The results in Table 2 show that the absorbed dose per organ for a CT scan ranges between 3.69 ± 0.58mGy (eyes) and 7.80 ± 1.18mGy (thyroid), while for CBCT, the doses are between 3.22 ± 0.51mGy (vertebrae) and 6.31 ± 1.02mGy (abdominal cavity). Most organs have slightly higher doses on CBCT compared to CT, especially central structures such as the brain (5.72 ± 0.92mGy vs. 4.92 ± 0.77mGy), brainstem (5.88 ± 0.95mGy vs. 5.02 ± 0.81mGy), liver (6.13 ± 0.99mGy vs. 5.67 ± 0.75mGy), or mandible. On the other hand, some organs located outside the main field receive a higher dose on CT, notably the thyroid (7.80mGy on CT vs. 3.61mGy on CBCT) and the heart (6.82mGy vs. 4.37mGy), which probably reflects a craniothoracic centering during the planning scan. These variations reflect the influence of acquisition geometry, organ position relative to the beam, and technical imaging parameters. Generally, the doses delivered by an isolated CT or CBCT remain low, but their repetition within the framework of the complete protocol justifies a cumulative evaluation, particularly for sensitive organs exposed recurrently.

**TABLE 2:** Absorbed doses from full‐body radiotherapy planning CT scan and CBCT.

Organs	Number of OSL holes in each organ	Absorbed dose (mGy)	
		Planning CT scan	CBCT
**Temporal and parietal bones**	4	4.81±0.62	4.786 ± 0.77
**Eyes**	2	3.69±0.58	4.227 ± 0.68
**Brain**	10	4.92±0.77	5.720 ± 0.92
**Brainstem**	2	5.02±0.81	5.878 ± 0.95
**Mandible**	3	5.53±0.06	6.132 ± 0.99
**Thyroid**	4	7.80±1.18	3.61 ± 0.58
**Sternum**	1	5.59±0.90	4.93 ± 0.79
**Heart**	5	6.82±0.98	4.373 ± 0.7
**Lungs**	12	5.69±0.89	5.651 ± 0.91
**stomach**	10	4.19±0.71	5.892 ± 0.95
**Liver**	18	5.67±0.75	6.129 ± 0.99
**spleen**	4	7.39±1.04	6.024 ± 0.97
**kidneys**	6	5.40±0.99	4.763 ± 0.77
**Abdominal cavity**	10	6.63±1.07	6.312 ± 1.022
**Vertebrates**	6	4.31±0.63	3.221 ± 0.51
**Bony pelvis**	14	4.40±0.67	6.131 ± 0.99
**Femoral heads**	2	5.68±0.83	5.817 ± 0.94
**Bladder**	6	5.37±0.79	5.783 ± 1.26
**Prostate**	1	5.32±0.88	4.935 ± 0.79
**Testicle**	2	4.19±0.81	4.610 ± 0.74

### 3.2 Dose estimates and organ evaluation in therapy and calculation of NTCP

#### 3.2.1 Organ dose assessment: Dosimetric comparison of 3D‐CRT versus VMAT techniques

**FIGURE 6:**
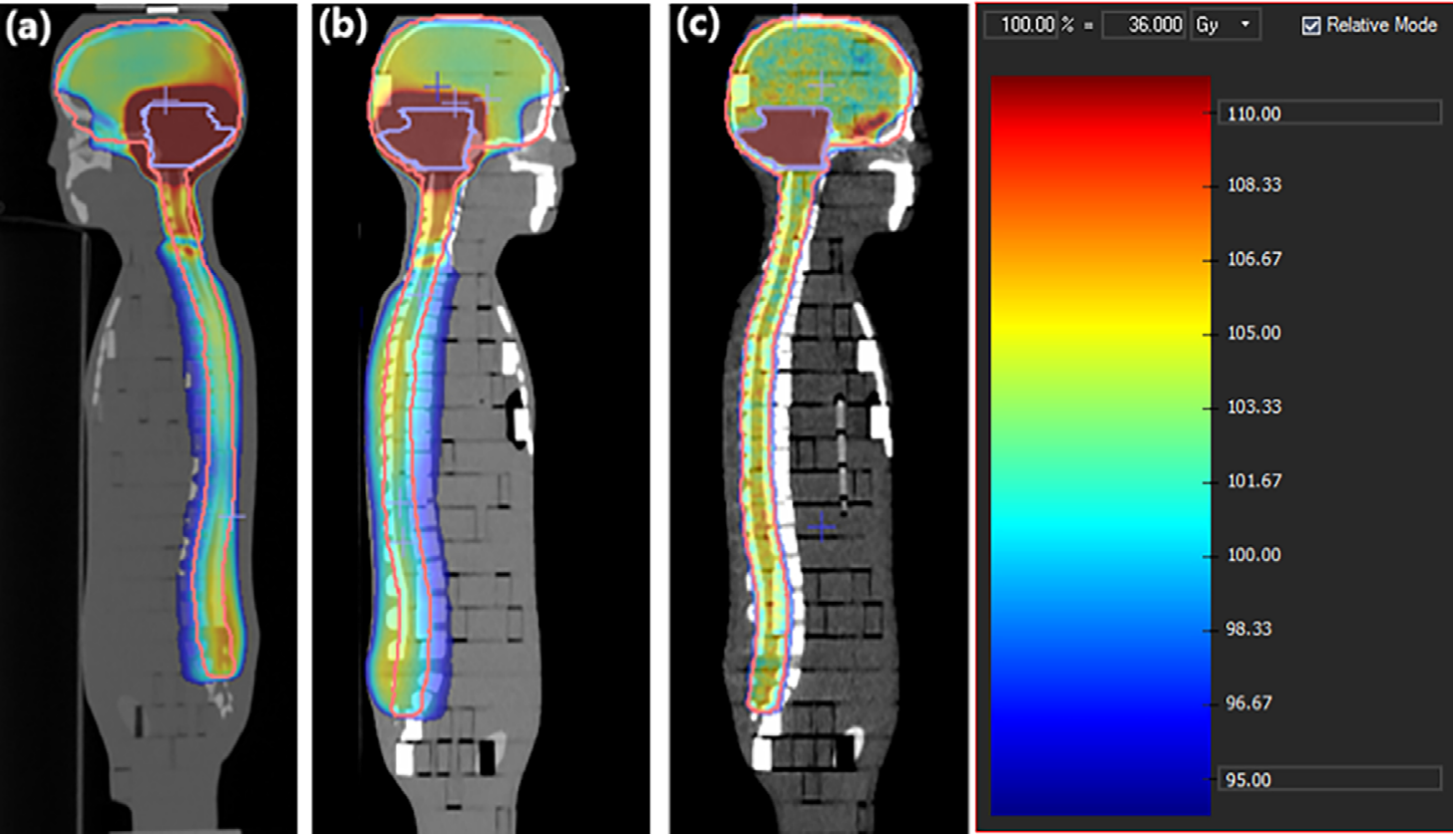
Distribution of 95% of the prescribed dose on the sagittal slice in (a) 3D‐CRT‐PP (b) 3D‐CRT‐SP (c) VMAT.

The results of our pediatric phantom dosimetric study show that VMAT provides a clear improvement in target coverage and preservation of organs at risk compared to 3D‐CRT, as shown in Figure 6 and Table 3. In agreement with the literature, the VMAT plan presents a more conformal dose to the PTV and a significant reduction of doses to normal organs. For example, the VMAT technique reduced the mean dose to the heart from approximately 16 Gy to 6.6 Gy, that to the thyroid from 27.1 Gy to 8.7 Gy, and that to the gonads from 8.0 Gy to 1.9 Gy, in contrast to 3D‐CRT. Similarly, our EUD maps indicate a notable reduction in exposure to many sensitive organs with VMAT: lenses (EUD reduced to ∼22 Gy in VMAT vs. ∼29–30 Gy in 3D‐CRT), lungs (∼10 Gy vs. ∼15–17 Gy), parotids (∼35 Gy vs. ∼41–43 Gy), and spinal cord (∼41 Gy vs. ∼45 Gy). However, structures directly adjacent to the tumor (brainstem, optic chiasm) maintain similar high doses between techniques, reflecting equivalent tumor coverage.

**TABLE 3:** Distribution of doses for different structures according to the three planning techniques.

Structures	3D‐CRT‐PP	3D‐CRT‐SP	VMAT
**Skin**	Dmax = 29.79 Gy	Dmax = 31.98 Gy	Dmax = 30.29 Gy
**Temporomandibular joint**	Dmax = 38.78 Gy	Dmax = 37.23 Gy	Dmax = 35.97 Gy
**Mandible**	Dmax = 23.89 Gy	Dmax = 23.92 Gy	Dmax = 28.679 Gy
**Brain**	V45 = 34.09% V50 = 25.83% V60 = 0%	V45 = 31.09% V50 = 26.48% V60 = 0%	V45 = 22.48% V50 = 23% V60 = 0%
**Brain steam**	Dmean = 53.95 Gy Dmax = 56.15 Gy	Dmean = 53.2 Gy Dmax = 57.43 Gy	Dmean = 53.1 Gy Dmax = 55.686 Gy
**Pituitary gland**	V40 = 100 Dmax = 52.63 Dmean = 32.89	V40 = 100 Dmax = 45.93 Dmean = 43.21	V40 = 100 Dmax = 45.93 Dmean = 31.53
**Right cochlea**	Dmax = 32.94 Gy Dmean = 31.67 Gy	Dmax = 34.94 Gy Dmean = 33.67 Gy	Dmax = 29.72 Gy Dmean = 30.74 Gy
**Left cochlea**	Dmax = 34.97 Gy Dmean = 35.93 Gy	Dmax = 35.47 Gy Dmean = 34.93 Gy	Dmax = 34.44 Gy Dmean = 33.94 Gy
**Left parotid**	Dmax = 29.40 V15 = 43.37% V45 = 0	Dmax = 28.24 V15 = 41.37% V45 = 0	Dmax = 29.76 Gy V15 = 0 V45 = 0
**Right parotid**	Dmax = 29.90 V15 = 64.21 V45 = 0	Dmax = 29.90 V15 = 62.35 V45 = 0	Dmax = 28.347 Gy V15 = 0 V45 = 0
**Retina**	Dmax = 38.32 Gy	Dmax = 39.78 Gy	Dmax = 37.12 Gy
**Oral cavity**	Dmean = 4.30 Gy Dmax = 11.85 Gy	Dmean = 3.30 Gy Dmean = 2.50 Gy	Dmax = 9.85 Gy Dmax = 4.9 Gy
**Thyroid**	Dmax = 31.23Gy Dmean = 25.97Gy	Dmax = 29.85 Gy Dmean = 23.21 Gy	Dmax = 25.041 Gy Dmean = 17.784 Gy
**Pharyngeal constrictor**	Dmax = 36.24 Gy Dmean = 29.40 Gy	Dmax = 37.91Gy Dmean = 29.2 Gy	Dmax = 32.58 Gy Dmean = 19.24 Gy
**Heart**	Dmax < 34.608 Gy V45 < 0% V60 < 0%	Dmax < 32.608 Gy V45 < 0% V60 < 0%	Dmax < 20.963 Gy V45 < 0% V60 < 0%
**Spinal cord**	Dmax = 41.89 Gy V57 < 0%	Dmax = 40.89 Gy V57 < 0%	Dmax = 39.89 Gy V57 < 0%
**Right lung**	V20 = 2.53% V30 = 0%	V20 = 3.53% V30 = 0%	V20 = 0.83% V30 = 0%
**Left lung**	V20 = 2.77% V30 = 0.16%	V20 = 3.77% V30 = 0.36%	V20 = 2.62% V30 = 0.01%
**Lungs**	V20 = 4.65% V30 = 0.20%	Dmax = 16.81 Gy V20 = 4.65% V30 = 0.20%	Dmax = 13.81 Gy V20 = 2.65% V30 = 0.20%
**Liver**	Dmax = 24.56 Gy	Dmax < 23.65	Dmax = 21.98
**Spleen**	Dmax = 2.96Gy Dmean = 0.73Gy	Dmax = 3.25Gy Dmean = 0.91Gy	Dmax = 2.14Gy Dmean = 0.87Gy
**Right kidney**	V20 = 15.20 V30 = 4.38 V50 = 0	V50 = 16.2% V30 = 5.27%	V50 = 0% V30 = 0.06%
**Left kidney**	V20 = 5.02% V30 = 4.39% V50 = 0	V50 = 16.32% V30 < 6.62%	V50 < 0% V30 < 0.02%
**Kidneys**	Dmean = 5.928 Gy	Dmean = 6.928 Gy	Dmean = 8.928 Gy
**Pancreas**	Dmax = 27.67 Dmean = 17.45	Dmax = 28.14 Dmean = 18.67	Dmax = 26.37 Dmean = 14.98
**Rectum**	Dmax = 34.39 Gy Dmean = 10.02 Gy	Dmax = 35.455 Gy Dmean = 11.045 Gy	Dmax = 28.12 Dmean = 9.51
**Abdominal cavity**	Dmax = 31.45 Gy	Dmax = 32.247 Gy	Dmax = 27.78 Gy
**Bladder**	Dmax = 28.59 Gy V70 = 0%	Dmax = 29.18 Gy V70 = 0%	Dmax = 15.97 Gy V70 = 0%
**Testicles**	Dmax = 0.71 Gy	Dmax = 0.61 Gy	Dmax = 0.26 Gy

**FIGURE 7:**
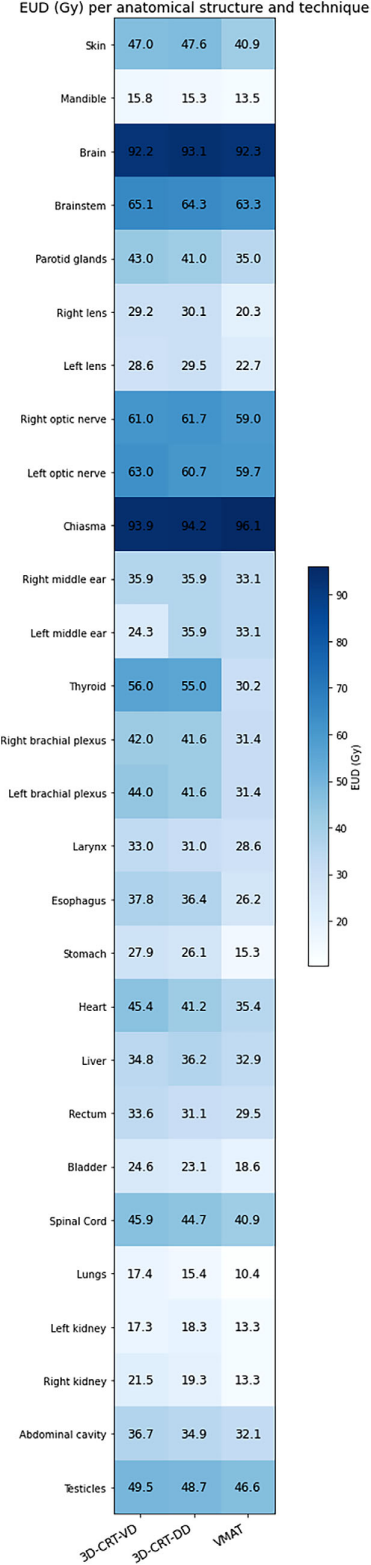
EUD heat maps (Gy) for various organs at risk according to the technique (3D‐CRT‐PP, 3D‐CRT‐SP, VMAT). Lighter colors reflect a lower equivalent dose to normal tissues with the VMAT technique.

It also appears that the two 3D‐CRT plans produce slightly different dose distributions to organs at risk. In our study, the 3D‐CRT‐PP configuration exposed some unilateral organs more than the 3D‐CRT‐SP plan, for example, the right kidney received an EUD of approximately 21.5 Gy in 3D‐CRT‐PP (vs. ∼19 Gy in 3D‐CRT‐SP and 13 Gy in VMAT), resulting in a renal NTCP equivalent to ∼21% with 3D‐CRT‐PP, much higher than < 1% with the other technique. Conversely, the left middle ear was less irradiated in 3D‐CRT‐PP (EUD ∼24 Gy) than in 3D‐CRT‐SP (∼36 Gy), suggesting that the 3D beam angle may selectively benefit certain organs. These discrepancies highlight the importance of careful planning even in 3D‐CRT to avoid unnecessary irradiation of some normal tissues. Nevertheless, the VMAT technique maintains overall the lowest doses across all non‐target organs, as evidenced by the widespread uptake of EUDs in the VMAT heat map, as shown in Figure 7.

#### 3.2.2 Calculation of NTCP

**FIGURE 8:**
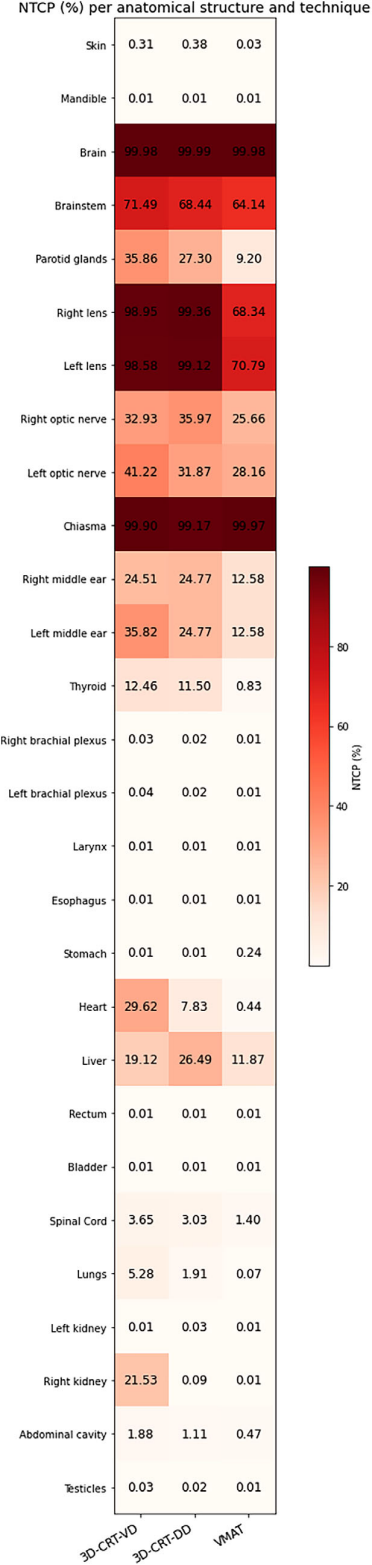
Probabilities of complications (NTCP, %) calculated for various organs at risk according to the technique. The reduction in NTCP (lighter color) with VMAT is notable for many organs (e.g., thyroid, heart, lens), reflecting a lower risk of probable effect.

The dose reduction to organs at risk achieved with VMAT translates into an expected reduction in the risk of radiation‐induced complications. According to our NTCP estimates (Figure 8), the probability of radiation‐induced thyroid dysfunction would decrease from approximately 11%–12% in 3D‐CRT to less than 1% with VMAT, thanks to the halving of thyroid EUD (Figure above). Similarly, the risk of cataract (crystalline NTCP ∼99% in 3D‐CRT) would be significantly reduced with VMAT (NTCP ∼70%), but not zero due to the persistence of a significant dose to the eyes. The predictive model also suggests a marked reduction in the risk of late cardiac death with VMAT (heart NTCP ∼0.4% vs. up to ∼30% in 3D‐CRT), consistent with the large reduction in mean myocardial dose recorded. Conversely, it should be noted that some structures retain high NTCP regardless of the technique, particularly the brain, optic chiasm, and optic nerves (NTCP ∼100% or > 90% in all cases), reflecting the inevitability of some influence in these organs given the high doses required for medulloblastoma. Finally, it should be emphasized that the VMAT technique, although more effective in sparing critical organs, inevitably exposes a larger body volume to low doses due to the diffuse radiation and the higher monitor unit (MU) multiplier. This “low‐dose bath” is a known pitfall of modulated techniques, associated with a theoretical excess risk of long‐term radiation‐induced cancers^35^. This is therefore a dosimetric compromise: VMAT optimizes the distribution of doses, particularly high doses to vital organs, at the cost of a slight increase in the tissue volume receiving low doses, a point to be taken into account in the long‐term monitoring of irradiated children.

## 4 DISCUSSION

The quantitative analysis of the absorbed doses of our results highlights that the contribution of the imaging steps (planning CT and CBCT) along the radiotherapy pathway remains very low compared to the total therapeutic dose delivered during the treatment. Indeed, the relative dose of a single CBCT examination generally represents less than 1% of the dose delivered by the therapeutic plans (3D‐CRT or VMAT), with percentages varying between 0.01 and 1.7% depending on the organs considered. The OSL measurements showed that the absorbed dose per organ for a single CBCT scan ranged from 3.2 to 6.3 mGy, depending on the anatomical region. In a realistic scenario, assuming 15 CBCT acquisitions over the entire treatment, the cumulative dose received is therefore between 48 mGy (vertebrae) and 95 mGy (abdominal cavity, bony pelvis, liver). When this dose is compared to the total prescribed therapeutic dose (i.e., 36 Gy for craniospinal irradiation and up to 54 Gy for tumor boost), for all organs at risk, the cumulative dose received by imaging (planned CT and 15 CBCT) systematically represents less than 0.3% of the prescribed dose to the target volume. For example, for the brain and brainstem, the total dose from CBCT amounts to 86 and 88 mGy, respectively, or only 0.24% of the prescribed dose (36 Gy) and 0.16% of the total dose with boost. Similarly, for the mandible (0.092 Gy for 15 CBCT vs. 23.9–28.7 Gy delivered in treatment), the ratio remains between 0.3% and 0.4% depending on the technique used. For peripheral organs with low exposure, such as the bladder or testes, the relative share of CBCT may appear high (e.g., 0.26% for the testes), but the absolute dose remains very low (< 0.1 Gy), which may be less clinically significant. For example, the thyroid receives a total imaging dose of approximately 0.06 Gy, or 0.17% of the therapeutic dose (36 Gy), while the dose achieved during treatment (VMAT) is 8.7 Gy, or 24% of the prescribed dose. Similarly, for the heart, the imaging dose represents 0.20% of the total dose, compared to 44% (3D‐CRT) or 18% (VMAT) for the therapeutic dose.

These proportions are found for the majority of organs analyzed, confirming that exposure to low doses generated by imaging, while not entirely negligible, remains minimal compared to the dominant role of treatment. These data are consistent with the results of the literature, which confirm that, even with a plan of 10 to 15 CBCTs per protocol, the increase in out‐of‐field dose related to positioning imaging remains negligible compared to the therapeutic dose, and well below the toxicity thresholds for normal tissue ^36^, ^37^ by having a cumulative imaging dose over the entire pediatric treatment protocol that generally varies between 0.1 and 0.5 Gy, that is, a biologically ineffective dose in an invasive tumor context^38^. However, in the specific context of pediatric medulloblastoma, this low dosimetric contribution of imaging may carry biological significance, as even low‐dose exposures in children have been associated with increased risks of leukaemia, brain tumours, and neurocognitive impairments.^39^

In this context, the daily use of CBCT for repositioning, although essential for therapeutic precision, represents a cumulative source of off‐target irradiation, especially for sensitive structures such as the spinal meninges. However, our results demonstrate that even with daily repeated CBCTs, the total dose received by the meninges remains negligible compared to therapeutic irradiation. This level of exposure has no therapeutic relevance: it does not contribute to the irradiation of the neuraxis and cannot induce a cytotoxic effect on disseminated tumor cells.

These results highlight the importance of focusing optimization and radioprotection efforts on treatment planning, while continuing to adjust imaging protocols. Although imaging doses represent only a minor fraction of the total therapeutic dose, their biological importance should not be underestimated in pediatric patients with medulloblastoma. The increased radiosensitivity of developing tissues, the cumulative effect of repeated acquisitions, and the long post‐treatment life expectancy of children present a significant long‐term risk, even with low‐dose exposure. Although the relative contribution of imaging to the total prescribed dose is small, its optimization remains essential in pediatric radiotherapy due to the cumulative radiosensitivity of developing tissues. Dose reduction strategies include pediatric adaptation of kVp and mAs, implementation of low‐dose CBCT protocols, limiting the frequency of imaging examinations through risk‐adapted IGRT schedules, and, where appropriate, the use of partial or targeted CBCT acquisitions. These measures further reduce cumulative exposure to imaging radiation while maintaining precise patient positioning, in accordance with the ALARA principle (As Low As Reasonably Achievable). With this in mind, our study was not limited to the dosimetric contribution of imaging, but also evaluated the clinical benefits of different treatment techniques by analyzing doses to healthy tissues and the probability of complications (NTCP), based on simulations of plans developed with various therapeutic approaches used in the management of pediatric medulloblastoma.

Our study found that the VMAT technique allows a significant reduction in the equivalent uniform dose (EUD) and the risk of complications (NTCP) in many organs at risk compared to 3D‐CRT techniques in the prone or supine position. This optimization is particularly marked in the thyroid, lenses, heart, kidneys, and spinal cord, consistent with data in the literature indicating better sparing of normal tissue by modulated techniques.^40^, ^41^ Despite this reduction, coverage of the target volume remains homogeneous in the brain and meningeal compartment, thus ensuring effective tumor control to prevent leptomeningeal relapses. This finding is particularly important because up to 80% of recurrences take the form of leptomeningeal metastases in this type of cancer ^40^, ^41^, ^42^.

The observed decrease in NTCP for key organs suggests an expected decrease in late side effects frequently reported in irradiated children, including endocrine deficits, hearing impairment, and cardiotoxicity ^43^, ^44^. However, some central structures, such as the brainstem, chiasm, or brain, retain high doses, resulting in NTCPs close to 100%, consistent with the high risk of neurocognitive sequelae already reported in several pediatric cohorts^43^. These results support international recommendations from the Children's Oncology Group (COG, North America) and the European Society for Paediatric Oncology (SIOPE, Europe), both of which provide clinical guidelines for pediatric medulloblastoma radiotherapy. Their recommendations emphasize: personalized treatment planning, with strict adaptation of techniques to age and risk group, use of conformal and intensity‐modulated techniques (IMRT, VMAT, proton therapy when available) to reduce exposure of healthy tissues, application of ALARA principles to limit cumulative doses, especially from imaging, and child‐friendly radioprotection, including minimization of scattered dose and safeguarding neurocognitive and endocrine functions ^40^, ^45^. Thus, the VMAT approach appears to be a relevant strategy to maximize the benefit‐to‐risk ratio, balancing tumor efficacy and functional preservation in the treatment of pediatric medulloblastoma. It is important to emphasize from this study, which dissects the pathway of the pediatric patient with medulloblastoma, that meningeal relapses are not related to imaging, but rather to the aggressive biology of medulloblastoma, its ability to spread through the cerebrospinal fluid (CSF), and to specifically underdosed irradiation zones. Therefore, even if daily imaging has become the standard in modern protocols to ensure proper positioning, it must be subject to continuous optimization (collimation, mAs, field of view) to limit cumulative exposure to normal tissue, particularly in children^46^.

Finally, these data highlight the need for rigorous post‐treatment neuroradiological monitoring, including regular brain and spinal magnetic resonance imaging (MRI) scans, to detect leptomeningeal relapse early, which remains a poor prognosis once established. Extended monitoring and the future integration of biomarkers (tumor deoxyribonucleic acid (DNA) circulating in the CSF, advanced imaging) constitute complementary avenues for improving relapse detection and early adaptation of management ^47^.

In conclusion, this study confirms the need for a detailed and personalized assessment of out‐of‐field doses in pediatric radiotherapy, combining experimental measurement and risk modeling. The systematic integration of the contribution of imaging, combined with the choice of compliant techniques, appears to be an essential strategy for maximizing the benefit/risk ratio, while strengthening the radiation protection of young patients with medulloblastoma. This integrated approach to dosimetry and risk, combining physical rigor and clinical awareness, paves the way for safer, personalized, and long‐term pediatric radiotherapy.

## 5 CONCLUSION

This study offers an integrated, quantitative, and experimental assessment of healthy tissue exposure throughout the radiotherapy pathway for pediatric medulloblastoma, using a pediatric anthropomorphic phantom, OSL dosimeters, and biological risk modeling tools. Our results demonstrate that the dosimetric contribution of imaging procedures (planning CT and monitoring CBCT) remains low relative to the prescribed therapeutic dose, consistently representing less than 0.3% of the total treatment dose and with no measurable impact on treatment delivery or deterministic toxicity.

Nevertheless, given the increased radiosensitivity of pediatric tissues and the cumulative nature of radiation exposure, even low imaging doses can contribute to long‐term stochastic risks. This underscores the importance of rigorous justification and optimization of imaging protocols, particularly in children.

However, the choice of treatment technique plays a crucial role in the dose received by organs at risk and the potential for late toxicity. Comparison between three‐dimensional conformal radiotherapy (3D‐CRT, in supine or prone position) and the VMAT technique demonstrated a significant reduction in EUD and NTCP for several critical organs with VMAT, including the thyroid, lens, heart, and kidneys, while maintaining optimal target coverage, an essential condition for controlling leptomeningeal recurrence. Some central structures remain exposed to higher doses, reflecting unavoidable anatomical constraints.

Overall, our results confirm the value of VMAT as a more advantageous therapeutic strategy for minimizing late toxicity while ensuring tumor control in pediatric radiotherapy. Above all, the originality of this work lies in the exhaustive evaluation of the cumulative exposure of healthy tissues at all stages of care, from treatment planning and image guidance to dose delivery, highlighting the need for integrated optimization strategies combining therapeutic and imaging practices to strengthen radioprotection in young patients.

## AUTHOR CONTRIBUTIONS

*Conceptualization*: Meriem Tantaoui and El Mehdi Essaidi. *Software*: Meriem Tantaoui. *Validation*: Mustapha Krim. *Formal analysis*: Meriem Tantaoui and Fatimzahra Chehab. *Investigation*: Meriem Tantaoui. *Writing—original draft*: Meriem Tantaoui. *Writing—review & editing*: Mohammed Reda Mesradi. *Visualization*: Abdelkrim Kartouni. *Supervision*: Abdelkrim Kartouni. *Project administration*: Souha Sahraoui. *Funding acquisition*: Mustapha Krim. All authors have read and agreed to the published version of the manuscript.

## CONFLICT OF INTEREST STATEMENT

The authors declare that they have no conflicts of interest.

## ETHICS STATEMENT

This study did not involve human participants or patient data and therefore did not require ethical approval.

## Data Availability

The data that support the findings of this study are available from the corresponding author upon reasonable request.
